# Les formes graves de la grippe A(H1N1) 2009 chez la femme enceinte: expérience du centre hospitalier universitaire de Fès, Maroc et revue de la littérature

**Published:** 2012-02-29

**Authors:** Mohamed Adnane Berdai, Smael Labib, Mustapha Harandou

**Affiliations:** 1 Service de réanimation mère et enfant, centre hospitalier universitaire Hassan II, Fès, Maroc

**Keywords:** Grippe, H1N1, Grossesse, Syndrome de détresse respiratoire aigu, Maroc

## Abstract

**Introduction:**

Le but de cette étude est de décrire les caractéristiques épidémiologiques, cliniques, paracliniques ainsi que l’évolution des femmes enceintes ou en post partum atteintes de formes graves de Grippe A(H1N1) 2009.

**Méthodes:**

C’est une étude prospective observationnelle monocentrique, menée au sein de notre service de réanimation mère et enfant au centre hospitalier universitaire Hassan II à Fès, sur une période de 3 mois, allant de novembre 2009 à janvier 2010.

**Résultats:**

L’âge moyen était de 28 ans, dans 85% des cas la grossesse se situaient au troisième trimestre, le syndrome grippal était constant, la SpO2 initiale était en moyenne de 86%. A la radiographie thoracique, un syndrome alvéolaire bilatéral était toujours présent. L’infection virale était confirmée dans tous les cas par la polymerase chain reaction. Chez 3 patientes la PaO2/FiO2 était inférieure à 300. L’Oseltamivir était l’antiviral utilisé chez toutes les parturientes. Un syndrome de détresse respiratoire aigu a été développé chez 28% des parturientes, elles ont été ventilées artificiellement avec des niveaux de pressions expiratoires positives à 14 +/- 1 cmH2O. L’évolution était favorable dans 71% des cas, cependant, 2 décès ont été déplorés.

**Conclusion:**

Les résultats rejoignent les données de la littérature, à savoir, un risque accru pour la femme enceinte de développer une forme grave, une présentation clinique similaire au reste de la population, l’intérêt de la vaccination et d’un traitement antiviral précoce et le rôle de l’ECMO dans le traitement des hypoxémies réfractaires.

## Introduction

Au début de 2009, un variant de la grippe A (H1N1) a fait son apparition au continent American, nommé initialement grippe porcine puis grippe A(H1N1) 2009. Ce virus résulte du réassortiment de deux souches Porcines dont l’une est une souche résultante d’un triple réassortiment Contenant des fragments originaires du virus humain H3N2, du virus de la grippe aviaire et de la grippe porcine [1]. L’Organisation mondiale de la santé (OMS) a déclaré l’état de Pandémie le 11 juin 2009. Les données provenant des précédentes pandémies du siècle passé (1918, 1957, 1968) montrent que les femmes enceintes encourent un risque accru concernant la mortalité et la morbidité par rapport au reste de la population [2]. Cette tendance semble se confirmer dans la pandémie de 2009, en effet la grossesse est confirmé comme facteur de risque de formes graves de grippe A(H1N1) [3].

Le but de cette étude est de décrire les caractéristiques épidémiologiques, cliniques, paracliniques ainsi que l’évolution des cas de femmes enceintes ou en post partum atteintes de formes graves de Grippe A(H1N1) admises au service de réanimation mère et enfant au CHU Hassan II de Fès.

## Méthodes

### Schéma de l’étude

Il s’agit d’une étude prospective observationnelle monocentrique, menée au sein de notre service de réanimation mère et enfant au Centre Hospitalier Universitaire Hassan II à Fès.

Nous avons considéré les cas de grippe A(H1N1)/2009 avérés selon les définitions de l’OMS et du Center for Disease Control and Prévention américain, à savoir toutes les femmes enceintes ou se présentant dans les 6 semaines suivant l’accouchement présentant un syndrome respiratoire aigu fébrile et une confirmation d’infection aigue à grippe A (H1N1)/2009 par real-time reverse transcriptase polymerase chain reaction (RT-PCR).

Les critères d’admission en réanimation étaient : une saturation pulsée en oxygène inférieure ( SpO2) à 93%, une polypnée supérieure à 30 cycles/minute, une tachycardie supérieure à 120 battements, une pression artérielle systolique inférieure à 90 mmHg, une hyperthermie ne répondant pas aux antipyrétiques, une hypothermie, des troubles de conscience, une suspicion de surinfection bactérienne respiratoire haute ou basse et tout autre signe clinique ou biologique de défaillance hémodynamique, neurologique, respiratoire, rénale, hématologique ou hépatique.

### Données collectées

Pour chaque cas suspect à l’entrée en réanimation, nous avons pratiqué une recherche de grippe A(H1N1)/2009 par RT-PCR sur le Prélèvement nasopharyngé (PNP) et lorsqu’elles étaient intubées, sur le prélèvement distal protégé (PDP).

L’âge, le terme de la grossesse, les comorbidités associées, la présentation clinique initiale, sont rapportés dans le Tableau 1. Le résultat des explorations radiologiques systématiques (radiographie du thorax, échocardiographie) et les principales données biologiques notamment la numération formule sanguine, la gazométrie, la C réactive protéine, la Troponine Ic sont rapportées dans le Tableau 2. La chronologie de la maladie, La prise en charge thérapeutique incluant les traitements anti-infectieux (antiviral et antibiotique), les détails de la ventilation mécanique, le recours aux autres traitements (drogues vaso-actives), ainsi que le devenir de la grossesse et l’évolution sont rapportes dans le Tableau 3.

**Tableau 1:** Données épidémiologiques et cliniques

Patiente	1	2	3	4	5	6	7	Moyenne
Age	32	36	24	27	25	26	28	28
Age gestationnel	28	28	25	33	22	36	J1 PP	28
Antécédents				RAA				
Délai (Spt- hosp)	3	7	7	4	6	3	1	4,4
Fièvre	+	+	+	+	+	+	+	100%
Sx respiratoires	+	+	+	+	+	+	+	100%
Myalgies	+	+	+	+	+	+	-	85%
Symptômes GE	+	-	+	+	-	-	+	42%
SpO2 initiale (%)	80	86	90	90	89	77	96	86

L’âge gestationnel exprimé en semaines d’aménorrhée. Post-partum : PP; RAA : rhumatisme articulaire aigu; Délai entre le début des symptômes et l’hospitalisation en jours: Délai (Spt- hosp); Sx : signes; Symptômes gastro-intestinaux : Symptômes GE ; Saturation pulsée en Oxygène: SpO2; + : présence du signe clinique ; - : absence du signe clinique.

**Tableau 2:** Données radiologiques et biologiques

Patiente	1	2	3	4	5	6	7	Moyenne
PaO2/FiO2	100	211	267	306	270	79	342	71% Pao2/FiO2<300
RT-PCR H1N1	+	+	+	+	+	+	+	100% Positive
Leucocytes	3,7	7,8	6,6	4,2	6	12,2	8,5	Hyperleucocytose :14%
Plaquettes	76	201	160	99	153	159	179	Thrombopénie: 28%
CRP	182	216	141	164	232	67	172	CRP> 100 : 85%
Troponine Ic	-	0,07	0,05	0,03	0,89	0,05	0,09	Positive : 14%
Opacités bilatérales	+	+	+	+	+	+	+	100%
Epanchement pleural	G	-	G	-	D	-	-	42%
Echocardiographie			RM					

PaO2/FiO2 : rapport entre la pression artérielle en oxygène et la fraction inspirée en oxygène. RT-PCR : real-time reverse transcriptase Polymerase Chain Reaction; Leucocytes en 10000 par mm3; Plaquettes en 10000 par mm3; CRP : C réactive protéine en mg/L; Opacités bilatérales : de type alvéolaire à la radiographie de thorax : si présent : (+); Epanchement pleural à la radiographie du thorax : absent: (-), présent à gauche : G, à droite : D; RM : rétrécissement mitral lâche, les autres patientes ont une échocardiographie normale

**Tableau 3:** Prise en charge thérapeutique et évolution

Patiente	1	2	3	4	5	6	7	Moyenne
Délai spt-trt	3	7	7	4	6	3	1	4,4 j
Oseltamivir	+	+	+	+	+	+	+	100%
B-Lactamines	+	+	+	+	+	+	+	100%
Macrolides	+	+	+	+	+	+	-	85%
Quinolones	-	-	-	-	-	+	+	28%
Corticothérapie	-	-	-	-	-	+	-	14%
Drogues vasoactives	-	-	-	-	-	+	-	14%
Ventilation non invasive	-	+	+	+	+	-	-	57%
Ventilation artificielle	+	-	-	-	-	+	-	28%
PEP	13					15		14
Durée de séjour en réanimation (j)	3	9	7	6	13	11	5	7,7
Durée de séjour des survivants (j)		9	7	6	13		5	8
Durée de séjour des décédés (j)	3					11		7
Devenir de la grossesse	MFIU	F	F	F	F	MNN J7	F	28% Défavorable
Evolution	Décès	F	F	F	F	Décès	F	71% Favorable

Délai entre début des symptômes et le début du traitement antiviral en journée : Délai spt-trt; (+): patiente ayant bénéficié du traitement ou du support ventilatoire; (-) : patiente n’ayant pas bénéficié du traitement ou du support ventilatoire; PEP : pression expiratoire positive en cmH2O; Durée de séjour : en journée (j); MFIU: Mort fœtale in utéro; MNN J7 : Mortalité néonatale au septième jour de vie; F : évolution favorable

## Résultats

Sept parturientes ont étés incluses dans cette étude, toutes présentant une forme grave de la grippe A(H1N1) avec confirmation biologique par RT-PCR, sur une période allant du 20/11/ 2009 au 15/01/2010.

### Données épidémiologiques et cliniques

L’étude a débuté le 25 novembre 2009 dès l’hospitalisation du premier cas de grippe A(H1N1)/2009 dans notre service, et s’est terminée le 30 janvier 2010.

Les données épidémiologiques et cliniques sont résumées dans le Tableau 1. La moyenne d’âge est de 28 ans (entre 22 et 36 ans) et l’âge gestationnel moyen a été de 28 semaines d’aménorrhée (SA) (entre 22 et 36 SA) ; six patientes ont été admises en pré-partum, cinq d’entre elle étaient au troisième trimestre de grossesse. Seulement une patiente a été admise en post-partum, et ce le lendemain de l’accouchement. Une seule patiente présentait une comorbidité associée à savoir un rhumatisme articulaire aigu. Le délai entre le début des symptômes et l’hospitalisation était de 4,4 jours (extrêmes de 1 à 7 jours).

La présentation clinique est dominée par le syndrome respiratoire aigu à début brusque, la fièvre et les signes respiratoires (toux, dyspnée, douleurs thoraciques) sont constants chez toutes les patientes. La saturation pulsée en oxygène à l’admission est en moyenne de 86% avec des extrêmes allant de 77 à 96%. En deuxième lieu survient les signes généraux (asthénie, myalgies, céphalées), les myalgies étaient présents chez 85% des cas. Les signes gastro-intestinaux étaient présents dans 42% des cas.

### Données paracliniques

Les données radiologiques et biologiques sont résumées dans le Tableau 2. Toutes Les patientes présentaient des opacités interstitiels et /ou alvéolaires bilatérales, associées à un épanchement pleural dans 42% des cas. L’échocardiographie cardiaque trans-thoracique a été réalisée chez toutes les parturientes pour éliminer une cardiopathie sous jacente et confirmer l’origine lésionnel d’un œdème aigu du poumon en cas de syndrome de détresse respiratoire aigu. Elle est sans particularités sauf pour une parturiente qui présentait un rétrécissement mitral lâche.

Les paramètres biologiques sont dominés par les résultats de la gazométrie artérielle qui montrent un rapport de PaO2/FIO2 inférieur à 300 dans 5 (71%) cas, dont deux inférieurs à 200 pour lesquelles la moyenne est de 90. La C réactive protéine était supérieure à 100 mg/l dans 85% des cas, une seule patiente présentait une hyperleucocytose, la thrombopénie était présente chez 28% des parturientes et la Troponine Ic positive dans un seul cas.

### Prise en charge et évolution

**Traitement:** Toutes les patientes ont bénéficié dès l’admission d’un traitement antiviral à base d’Oseltamivir (Tamiflu ®) à la dose de 75 milligrammes/jour en deux prises par voie orale et ce durant cinq jours. Le délai entre le début des symptômes et l’instauration du traitement antiviral était en moyenne de 4,4 jours (extrêmes allant de 1à7 jours). Lorsque la patiente était en trouble de conscience ou intubé le produit était administré par sonde nasogastrique. Dans tous les cas un traitement antibiotique probabiliste a été instauré en prépartum à base de Beta lactamines (Amoxicilline+ acide clavulanique ou Ceftriaxone) associés à un macrolide (Rovamycine). En post partum, c’est une quinolone de deuxième génération (Ciproxine) qui est associée aux mêmes Beta lactamines.

Pour ce qui est des traitements adjuvants ; La corticothérapie a été administré chez une seule patiente à raison de 200 milligrammes d’hemisuccinate d’hydrocortisone par jour en 4 prises. Dans un cas, on a eu recours aux drogues vasoactives à base de Noradrénaline. La prescription de cimétidine était systématique, la thromboprophylaxie était assuré par Enoxoparine sauf dans un cas de thrombopénie ou on s’est limité aux moyens mécaniques.

**La ventilation non invasive et mécanique:** Les parturientes qui ne présentaient ni Acute lung injury (ALI), ni syndrome de détresse respiratoire aigu (SDRA) étaient au nombre de deux, l’une d’elles a bénéficié de la ventilation non invasive (VNI) avec bonne évolution, alors que la deuxième a bénéficié d’une oxygénation uniquement au masque à haute concentration. Trois patientes ont présenté un ALI qui a bien évolué sous VNI sans recours à l’intubation trachéale.

Les deux patientes ayant présenté un SDRA ont été ventilées artificiellement rapidement après l’échec de la VNI. La ventilation a requis des niveaux élevés de pression expiratoire positive (PEP) qui était en moyenne de 14 cmH2O, sans barotraumatisme associé. Pour ce qui de l’évolution fœtale, on a recensé un cas de mort fœtale in utero parmi les 6 cas de grossesse évolutive et un cas de mortalité néonatale au septième jour de vie, à la suite d’une césarienne pour souffrance fœtale aigu chez une parturiente présentant un SDRA. Dans les autres cas l’accouchement s’est déroulé sans particularité à distance de l’épisode infectieux. La durée de séjour en réanimation était de 7,7 jours (allant de 3 à 13 jours), l’évolution est favorable sans séquelles dans 5 cas (71%), par contre on a déploré le décès de deux patientes, l’une par choc septique sur surinfection bactérienne et l’autre dans un tableau d’hypoxémie réfractaire.

## Discussion

Les femmes enceintes sont particulièrement vulnérables face à la grippe pandémique A(H1N1) 2009. Elles présentent un risque accru de développer une forme grave de la maladie par rapport à la population générale [4] En Australie et en nouvelle Zélande que la femme enceinte présente environ sept fois plus de risque d′admission en unité de soins intensifs par rapport aux femmes non enceintes en âge de procréer [5]. Cette vulnérabilité particulière des femmes enceintes aux sous-types A (H1N1) a déjà été rapportée lors de pandémies précédentes de ce sous-type viral [6,7]. Plusieurs hypothèses physiopathologiques viennent expliquer ce fait ; d’abord les femmes enceintes sont une population jeune nées après la pandémie de 1957, les patients ayant été en contact lors de cette épidémie avec le virus circulant possèdent une immunité croisé les protégeant des formes graves de la pandémie actuelle [8], les modifications immunologiques lors de la grossesse peuvent à leur tour expliquer l’incidence élevée des formes graves, la réponse immunitaire à médiation cellulaire étant déviée vers une réponse à médiation humorale pour s’adapter a la greffe semiallogenique que constitue le fœtus [9,10], d’autre part, la grossesse confère une tolérance immunitaire particulière aux antigènes d’origine paternels du fœtus, cette condition pourrait être responsable d’une protection immunitaire diminuée de la femme enceinte vis-à-vis du virus de la grippe, et surtout vis-à-vis des surinfections bactériennes pulmonaires dans ce contexte [11]. Un autre déterminant est les modifications physiologiques survenues lors de la grossesse, ainsi, la compression de voisinage imposée par l’utérus gravide gêne la fonction respiratoire en diminuant les volumes résiduels fonctionnels et la compliance thoracique [12], sur le plan cardiovasculaire la surcharge volumique peut rapidement limiter les capacités d’adaptation hémodynamiques à une condition aiguë de sepsis sévère [13]. Dans notre série une seule parturiente présentait une comorbidité associée à savoir un rhumatisme articulaire aigu sans atteinte cardiaques, les autres ayant développé une forme grave de la grippe A(H1N1) avec comme seul facteur de risque la grossesse. Un seul cas est admis au deuxième trimestre de grossesse, 5 cas au troisième trimestre et un en post-partum immédiat, cela se concorde avec la littérature qui montre une incidence élevée de cas graves au cours du troisième trimestre. Dubar et al [14], à travers le registre national français de femmes enceintes ayant une grippe A(H1N1) confirmée biologiquement, a recensé 40 cas admis en unité de soins intensifs, 70% d’entres elles étaient au troisième trimestre ou en post-partum, Louie et al [15] dans une étude américaine a montré que 57% des patientes hospitalisées ou décédées étaient au troisième trimestre.

La présentation clinique initiale de la grippe A(H1N1) chez la femme enceinte est généralement similaire à celle rapportée par le reste de la population, Jamieson et al dans une étude américaine concernant les femmes enceintes atteintes par la pandémie de grippe de 2009 ont rapporté une symptomatologie clinique initiale dominé par la fièvre(97%), la toux(94%), la rhinorhée ( 59%), mal de gorge (50%),par la suite, les céphalées, la dyspnée, les myalgies, les vomissements, la diarrhée et la conjonctivite [16]. Les résultats de notre série vont dans le même sens concernant la fièvre et les signes respiratoires qui sont retrouvés dans tous les cas, suivi des signes généraux (myalgie 85%) et des signes gastro-intestinaux dans 42% des cas. D’autres manifestations plus rares ont été décrites chez la population générale il s’agit de péricardites, de myocardites et de rhabdomyolyses [15], Erden et al [17] ont démontré une association entre les patients hospitalisés pour la grippe A(H1N1) 2009 et un dysfonctionnement cardiaque infraclinique. Dans notre série l’échocardiographie réalisée chez toutes les patientes n’a révélé pas d’atteinte cardiaque due au virus de la grippe A (H1N1).

L’imagerie thoracique des patients admis en réanimation montre dans trois quart des cas un infiltrat interstitiel ou alvéolaire prédominant en bibasal [1], cet infiltrat était présent chez toutes nos patientes avec en plus un épanchement pleural dans 3 cas.

La vaccination par un vaccin fragmenté sans adjuvant est recommandée aux femmes enceintes à partir du deuxième trimestre de la grossesse. Cette vaccination n’est pas recommandée au cours du premier trimestre de la grossesse, sauf facteur de risque surajouté [4]. La vaccination des femmes enceintes devrait théoriquement conférer une protection passive par les anticorps maternels aux nouveaux nés après la naissance [18]. Aucune parturiente de notre série n’a bénéficié de vaccination préventive au cours de sa grossesse contre le virus de la grippe A(H1N1) 2009, et ce, malgré les recommandations dans ce domaine, ce qui peut expliquer entre autre l’apparition de formes graves chez ces patientes. Le traitement repose sur l’administration d’antiviraux, les deux molécules connues efficaces contre le virus de la grippe A (H1N1) 2009 sont l’Oseltamivir (Tamiflu®) par voie orale et le Zanamivir (Relenza®) par voie inhalé, ce sont des inhibiteurs de la neuraminidase qui empêchent le virus de traverser la membrane plasmique de la cellule hôte infectée. La sureté de ces molécules lors de la grossesse est constatée lors de leurs utilisations dans la grippe saisonnière [19]. Le bénéfice du traitement par ces molécules semble dépasser les risques pour le fœtus [16]. Chez la femme enceinte, L’Oseltamivir est préféré au Zanamivir, du fait d’un recul plus important dans cette tranche de population [20], de plus il a un passage placentaire faible, de 15% à 20% chez l’animal et probablement moins chez l’homme, et n’a d’effets secondaires connus que des nausées et vomissements [21]. La posologie usuelle était de 75 milligrammes par voie orale deux fois par jour pendant 5 jours, certaines équipes proposent de doubler la dose et d’allonger le traitement à 10 jours dans les formes graves [4,22]. Les recommandations de l’OMS est de traiter systématiquement les femmes enceintes dès le début des symptômes [23]. Le traitement antiviral doit être débuté dans les 48 heures suivant l’apparition des symptômes cliniques [24]. Le Center for Disease Control (CDC) avait recommandé de commencer les antiviraux sur des conclusions cliniques, sans être guidé ou retardé par les résultats du rapid test, Dans la série de Louie et al, les femmes enceintes ayant reçu ce traitement 48 heures après le début des symptômes avaient un risque 4 fois plus élevé d’être admises en unité de soins intensifs ou de mortalité que celles qui ont reçues le traitement tardivement [25]. Dubar et al ont retrouvé une association forte entre une évolution sévère et le retard du début de traitement par l’Oseltamivir après le début des symptômes (> 3 ou 5 jours) [14].

Dans notre série, Toutes les patientes ont bénéficié dès l’admission d’un traitement antiviral à base d’Oseltamivir (Tamiflu®) à la dose de 75 milligrammes/jour en deux prises par voie orale et ce durant cinq jours. Le délai entre le début des symptômes et l’instauration du traitement antiviral était en moyenne de 4,4 jours. Une seule patiente a bénéficié du traitement antiviral dans les 48 heures suivant l’apparition des symptômes, l’évolution était favorable pour elle et son fœtus. Ce retard pourra être expliqué par la difficulté d’accès aux soins, la présence de certains symptômes attribués à la grossesse plutôt qu’a une éventuelle atteinte grippale et la réticence à prescrire des antiviraux chez une femme enceinte.

Le traitement antibiotique en association au traitement antiviral se discute chez la femme enceinte en cas de forme grave et, dans tous les cas, à la moindre suspicion de surinfection bactérienne, cela d’autant plus que le tableau clinique est sévère ou la prise en charge est tardive [4]. Toutes nos patientes ont reçu un traitement antibiotique probabiliste à base de beta lactamines associées en pré-partum à un macrolide et en post-partum à une quinolone, notre attitude est expliquée par la gravité du tableau clinique initiale et le retard de prise en charge thérapeutique, ainsi que la crainte d’une aggravation de l’état de nos patientes.

La corticothérapie est utilisée par certaines équipes dans les formes graves de la grippe A(H1N1) 2009 associées à un SDRA, elle est instituée selon les séries chez 18 à 100% des patients, les produits utilisés étaient l’hydrocortisone à raison de 300 mg/jour ou la méthylprednisolone 60 mg/jour [1]. Quispe-Laime et al ont rapportés que chez des patients présentant un SDRA avec ou sans confirmation de la grippe H1N1, une dose prolongée faible à moyenne de corticostéroïdes est bien toléré et associée à une amélioration significative des lésions pulmonaires, des scores de défaillances multiviscérales et une mortalité hospitalière basse [26]. Cependant, Plusieurs équipes étaient réticentes à l’utilisation de corticostéroïdes dans les formes graves s’alignant ainsi sur les recommandations de l’oms de peur de modifier la charge virale [27]. Ces données concernent la population générale et ne sont pas spécifiques à la femme enceinte. Dans notre pratique, du fait de l’absence de consensus, on n’a eu recours à la corticothérapie que dans un seul cas de choc septique suite à une surinfection bactérienne pulmonaire.

La présentation classique des infections sévères due au virus H1N1 2009 est dominée par l’atteinte respiratoire, la prise en charge de cette défaillance respiratoire de la femme enceinte doit obéir à deux impératifs, d’une part, s’adapter aux modifications physiologiques de la grossesse et, d’autre part, maintenir une oxygénation fœtale adéquate.

La ventilation non invasive(VNI) a une place dans la prise en charge ventilatoire des formes graves de la grippe A (H1N1), parmi les 40 parturientes admise en unité de soins intensifs en France durant la pandémie, cinq d’entre elles ont bénéficié de la VNI avec bonne évolution [14]. Son utilisation dans le SDRA reste sujet de controverse, l’utilisation de la ventilation non invasive dans la grippe pandémique 2009 associée au SDRA a été rapportée dans plusieurs séries [28,29,30,31], le taux d’échec était le plus souvent élevé (> ;75%) motivant l’intubation assez rapidement [1], Djibré l’a utilisée avec succès dans le traitement d’une femme enceinte atteinte d’un SDRA associée à la grippe A H1N1 [32]. Nous l’avons utilisé dans quatre cas, dont trois présentait un ALI avec un rapport PaO2/FiO2 inférieur à 300, la VNI leur a permis d’éviter l’intubation trachéale avec une bonne évolution.

En général, et surtout en cas de SDRA sévère chez la femme enceinte, l’approche ventilatoire est basée sur une intubation trachéale prévue, du fait qu’une intubation urgente pourrait avoir des conséquences dévastatrices sur la mère et le fœtus et qu’elle nous permet d’instaurer une ventilation protectrice [33]. Dans une étude collectant les données de 1131 Patients provenant de 7 pays, atteints de SDRA associé à une grippe A(H1N1), 65 et 90 % des patients ont nécessité une ventilation mécanique invasive. Cette ventilation était souvent difficile avec des hauts niveaux de FiO2 et de pression expiratoire positive (PEP).Les pressions de plateau étaient également élevées. Les barotraumatismes étaient rapportes entre 10 et 15 % des cas [1].

Une revue de la littérature concernant les femmes enceintes atteintes de grippe A H1N1 et admise en unité de soins intensifs montre un taux élevé de femmes intubées, il est de 66% (77/115 cas) aux Etats unis [34], 50% (20/40 cas) en France [14], 69% (44/ 64 cas) en Australie et nouvelle Zélande [3]. Parmi nos patientes, deux (28%) ont présenté un SDRA et ont été donc intubées et ventilées, les paramètres de la ventilation sont été basés sur les recommandations sus citées, on a eu recours à un niveau de PEP élevé qui était de 14 cmH2O en moyenne et on n’a pas noté de barotraumatismes. Malheureusement, les deux patientes sont décédées, l’une suite à un choc septique sur surinfection bactérienne de sa pneumopathie, l’autre dans un tableau de défaillance respiratoire majeure vu le manque de moyens thérapeutiques alternatifs, notamment l’extracorporeal membrane oxygénation (ECMO), cette technique est utilisée dans le cas du traitement de l’hypoxémie réfractaire résultant d’un SDRA, après l’échec la ventilation mécanique associée aux thérapies adjuvantes usuelles (décubitus ventral, oxyde d’azote). L’ECMO est un support extracorporel de la respiration qui peut être conduit de façon saine, permettant aux poumons de se mettre au repos et de guérir et aussi d’éviter la Ventilation Induced Lung Injury (VILI) [35]. En France, parmi les 20 patientes ayant requises la ventilation mécanique, onze ont eu recours à l’ECMO pour une durée moyenne de 8 jours, neuf d’entre elle ont survécu [14]. Ces résultats bien qu’établis à partir d’échantillons réduits montre l’intérêt de l’ECMO dans le traitement de la défaillance respiratoire dans le cas du SDRA, et nous incite à se doter du plateau technique et humain pour réaliser cette technique dans notre formation.

Il existe une incertitude sur le moment optimal et le mode d’accouchement des femmes enceintes porteuses d’une défaillance respiratoire. L’effet de la grossesse sur la physiologie respiratoire de la mère, laisse suggérer que la délivrance de la patiente enceinte présentant une insuffisance respiratoire se traduira par une amélioration de l′ état de la mère, certains auteurs ont conclu que la césarienne offre un meilleur pronostic dans la gestion de l’hypoxie maternelle [3], les publications qui supportent cette idée sont limitées. L’accouchement par césarienne indiqué par l’hypoxie maternelle est généralement non recommandé dans la littérature puisqu’il n’améliore pas le pronostic maternel [36]. L’indication d’un accouchement doit donc d’abord être fœtale (fœtus viable et hypoxie maternelle sévère) avant d’être maternelle, l’accouchement ne devant pas être réalisé dans le seul but d’améliorer l’oxygénation maternelle. Les données publiées sont cependant relativement pauvres et les décisions doivent être prises au cas-par-cas [4]. La voie d’accouchement sera alors déterminée par les indications obstétricales [37]. Notre série comporte deux accouchements qui se sont déroulés pendant la phase aigue de l’infection grippale, les autres se sont déroulées à distance de l’épisode infectieux et se sont déroulées sans particularité. L’un des deux cas est une parturiente admise en post-partum immédiat et qui a accouché par voie basse avant l’hospitalisation, avec une bonne évolution. L’autre cas est une parturiente en SDRA césarisée à 36 semaines d’aménorrhée pour souffrance fœtale aigu, pourtant, la césarienne n’a pas amélioré sa fonction respiratoire, l’évolution été marqué par le décès maternelle, cela conforte les données précitées qui indiquent que malgré la possibilité de l’amélioration de l’oxygénation maternelle par l’accouchement, le stress chirurgical pourra l’aggraver.

L′infection par le virus A(H1N1) de 2009 a eu un impact substantiel sur les résultats néonatals. La plupart des nouveau-nés de femmes gravement malades dans L’étude australienne ont été admis dans une unité néonatale spéciale, comparativement à moins de 15% de la population totale. Cependant, il est actuellement inconnu, si un accouchement prématuré chez des femmes de plus de 30 semaines d’aménorrhée pourra améliorer le pronostic néonatal [3]. Dans une série américaine regroupant 788 femmes enceintes atteintes de grippe A(H1N1)2009, chez les femmes parmi lesquelles les données sur l’évolution de la grossesse ont été disponibles, le taux de prématurité était de 30,2%, qui est plus élevé que le taux de naissances prématurées établie à 13% à l′échelle nationale. Ces données bien qu’ils ne sont pas concluantes, sont compatibles avec les données suggérant un taux élevé d’accouchement prématuré au cours des précédentes pandémies [34]. Seul l’étude Française échappe à ce constat, elle montre un taux de mortalité et de morbidité extrêmement bas, ces données ont été collectées à partir d′une grande série de bébés (146) livré par les mères qui avaient été traités par l′Oseltamivir, renforçant ainsi des données déjà publiées sur l′innocuité de ce médicament antiviral pendant la grossesse [14,38]. Dans notre expérience, on a constaté un cas de mort fœtale in utero, et un décès au septième jour de vie suite à une césarienne. Au total, sur les sept grossesses initiales, cinq (71%) ont bien évolué donnant naissance à des nouveaux nés bien portants.

La contamination des nouveau-nés par leur mère se fait de façon quasi exclusive par voie aérienne en post-partum. De ce fait, les mesures préventives pendant l’accouchement et un isolement de la maman grippée dès la naissance de l’enfant devraient permettre de limiter la contamination [39].

## Conclusion

Bien que le nombre de cas inclus dans cette étude soit réduit, les résultats rejoignent les données de la littérature mondiale, à savoir, un risque accru pour la femme enceinte de développer une forme grave, une présentation clinique similaire au reste de la population, l’intérêt de la vaccination et d’un traitement antiviral précoce et le rôle de l’ECMO dans le traitement des hypoxémies réfractaires.

## References

[1] Jaber S, Conseil M, Coisel Y, Jung B, Chanques G (2010). ARDS and influenza A (H1N1): patients’ characteristics and management in intensive care unit. A literature review. Ann Fr Anesth Reanim.

[2] May Li Lim, Wai Yee Lim, Nancy WS Tee, Siok Hong Lim, Jing Jye Chee (2010). Obstetric Outcomes of Influenza A H1N1 (2009) Infection in Pregnancy-Experience of a Singapore Tertiary Hospital. Ann Acad Med Singapore.

[3] ANZIC Influenza Investigators and Australasian Maternity Outcomes Surveillance System (2010). Critical illness due to 2009 A/H1N1 influenza in pregnant and postpartum women: population based cohort study. BMJ.

[4] Dubar G, Launay O, Batteux F, Tsatsaris V, Goffinet F, Mignon A (2010). Pregnancy and pandemic influenza A(H1N1) 2009. Current concepts for anaesthesia and critical care medicine. Ann Fr Anesth Reanim.

[5] Lapinsky SE (2010). Critical illness as a result of influenza A/H1N1 infection in pregnancy. BMJ.

[6] Mullooly JP, Barker WH, Nolan TF (1986). Risk of acute respiratory disease among pregnant women during influenza A epidemics. Public Health Rep.

[7] McKinney WP, Volkert P, Kaufman J (1990). Fatal swine influenzapneumonia during late pregnancy. Arch Intern Med..

[8] Chowell G, Bertozzi SM, Colchero MA, Lopez-Gatell H, Alpuche-Aranda C, Hernandez M (2009). Severe respiratory disease concurrent with the circulation of H1N1 influenza. New Eng J Med..

[9] Maggi E, Parronchi P, Manetti R, Simonelli C, Piccinni MP, Rugiu FS, De Carli M, Ricci M, Romagnani S (1992). Reciprocal regulatory effects of IFN-gamma and IL-4 on the in vitro development of human Th1 and Th2 clones. J Immunol.

[10] Hsieh CS, Macatonia SE, Tripp CS, Wolf SF, O’Garra A, Murphy KM (1993). Development of TH1 CD4+ T cells through IL-12 produced by Listeria-induced macrophages. Science.

[11] Stegemann S, Dahlberg S, Kroger A, Gereke M, Bruder D, Henriques-Normark B (2009). Increased susceptibility for superinfection with Streptococcus pneumoniae during influenza virus infection is not caused by TLR7-mediated lymphopenia. PloS One..

[12] Yeomans ER, Gilstrap LC (2005). Physiologic changes in pregnancy and their impact on critical care. Crit Care Med.

[13] Rodrigues J, Niederman MS (1992). Pneumonia complicating pregnancy. Clin Chest Med..

[14] Dubar G, Azria E, Tesnière A, Dupont H, Le Ray C, Baugnon T, Matheron S, Luton D, Richard JC, Launay O, Tsatsaris V, Goffinet F, Mignon A (2010). French Registry on 2009 A/H1N1v during pregnancy. French Experience of 2009 A/H1N1v Influenza in Pregnant women. PLoS One.

[15] Louie JK, Acosta M, Jamieson DJ, Honein MA (2010). the California Pandemic (H1N1) Working Group. Severe 2009 H1N1 Influenza in Pregnant and Postpartum Women in California. N Engl J Med.

[16] Denise J Jamieson, Margaret A Honein, Sonja A Rasmussen (2009). H1N1 2009 influenza virus infection during pregnancy in the USA. Lancet.

[17] Ismail Erden, Emine Cakcak Erden, Hakan Ozhan (2010). Echocardiographic manifestations of pandemic 2009 (H1N1) influenza a virus infection. J Infect.

[18] Reuman PD, Ayoub EM, Small PA (1987). Effect of passive maternal antibody on influenza illness in children: a prospective study of influenza A in mother-infant pairs. Pediatr Infect Dis J.

[19] Boon H Lim, Tahir A Mahmood (2010). Pandemic H1N1 2009 (swine flu) and pregnancy. Obstetrics,gynaecology and reproductive medecine.

[20] Collége national des gynecologues et obstétriciens francais (2009). Conduite à tenir pour les femmes enceintes en cas d’épidémie de grippe A (H1N1). http://wwwcngofassofr.

[21] Picone O, Ami O, Vauloup-Fellous C, Martinez V, Guillet M, Dupont-Bernabé C, Donnadieu AC, Trichot C, Senat MV, Fernandez H, Frydman R (2009). Pandemic influenza A H1N1 2009 flu during pregnancy: Epidemiology, diagnosis and management. J Gynecol Obstet Biol Reprod (Paris).

[22] Saleeby E, Chapman J, Morse J, Bryant A (2009). H1N1 influenza in pregnancy: cause for concern. Obstet Gynecol.

[23] WHO (2009). Pandemic influenza in pregnant women. Pandemic (H1N1) 2009 briefing note 5.

[24] Centers for Disease Control and Prevention

[25] Louie JK, Acosta M, Jamieson DJ, Honein MA (2010). California Pandemic (H1N1) Working Group. Severe 2009 H1N1 influenza in pregnant and postpartum women in California. N Engl J Med.

[26] Quispe-Laime AM, Bracco JD, Barberio PA, Campagne CG, Rolfo VE, Umberger R, Meduri GU (2010). H1N1 influenza A virus-associated acute lung injury: response to combination oseltamivir and prolonged corticosteroid treatment. Intensive Care Med..

[27] Confalonieri M, D’Agaro P, Campello C (2010). Corticosteroids do not cause harmful increase of viral load in severe H1N1 virus infection. Intensive Care Med.

[28] Miller RR, Markewitz BA, Rolfs RT, Brown SM, Dascomb KK, Grissom CK, Friedrichs MD, Mayer J, Hirshberg EL, Conklin J, Paine R, Dean NC (2010). Clinical findings and demographic factors associated with ICU admission in Utah due to novel 2009 influenza A(H1N1) infection. Chest..

[29] Rello J, Rodriguez A, Ibanez P, Socias L, Cebrian J, Marques A (2009). Intensive care adult patients with severe respiratory failure caused by influenza A(H1N1)v in Spain. Crit Care..

[30] Domínguez-Cherit G, Lapinsky SE, Macias AE, Pinto R, Espinosa-Perez L (2009). Critically Ill patients with 2009 influenza A(H1N1) in Mexico. JAMA.

[31] Kumar A, Zarychanski R, Pinto R, Cook DJ, Marshall J, Lacroix J (2009). Critically ill-patients with 2009 influenza A(H1N1) infection in Canada. JAMA.

[32] Djibré M, Berkane N, Salengro A, Ferrand E, Denis M, Chalumeau-Lemoine L, Parrot A, Mayaud C, Fartoukh M (2010). Non-invasive management of acute respiratory distress syndrome related to Influenza A (H1N1) virus pneumonia in a pregnant woman. Intensive Care Med.

[33] Cabrini L, Silvani P, Landoni G, Moizo E, Colombo S, Zangrillo A (2010). Noninvasive ventilation in H1N1-correlated severe ARDS in a pregnant woman: please, be cautious!. Intensive Care Med.

[34] Siston AM, Rasmussen SA, Honein MA, Fry AM, Seib K, Callaghan WM, Louie J (2010). Pandemic 2009 influenza A(H1N1) virus illness among pregnant women in the United States. JAMA.

[35] Bessereau J, Chenaitia H, Michelet P, Roch A, Gariboldi V (2010). Acute respiratory distress syndrome following 2009 H1N1 virus pandemic: when ECMO come to the patient bedside. Ann Fr Anesth Reanim.

[36] Tomlinson MW, Caruthers TJ, Whitty JE, Gonik B (1998). Does delivery improve maternal condition in the respiratory-compromised gravida?. Obstet Gynecol.

[37] Stephan E Lapinsky (2010). H1N1 Novel influenza A in pregnant and immune-compromised patients. Crit Care Med.

[38] Greer LG, Sheffield JS, Rogers VL, Roberts SW, McIntire DD, Wendel GD (2010). Maternal and neonatal outcomes after antepartum treatment of influenza with antiviral medications. Obstet Gynecol.

[39] Grimpel E (2009). Pandémie grippale et organisation des soins en obstétrique et néonatologie.

